# Identifying unfamiliar voices: Examining the system variables of sample duration and parade size

**DOI:** 10.1177/17470218231155738

**Published:** 2023-03-07

**Authors:** Nikolas Pautz, Kirsty McDougall, Katrin Mueller-Johnson, Francis Nolan, Alice Paver, Harriet M. J. Smith

**Affiliations:** 1 Nottingham Trent University, Nottingham, UK; 2 University of Cambridge, Cambridge, UK; 3 University of Oxford, Oxford, UK

**Keywords:** Earwitness, voice parades, voice identification, witness memory, system variables

## Abstract

Voice identification parades can be unreliable due to the error-prone nature of earwitness responses. UK government guidelines recommend that voice parades should have nine voices, each played for 60 s. This makes parades resource-consuming to construct. In this article, we conducted two experiments to see if voice parade procedures could be simplified. In Experiment 1 (*N* = 271, 135 female), we investigated if reducing the duration of the voice samples on a nine-voice parade would negatively affect identification performance using both conventional logistic and signal detection approaches. In Experiment 2 (*N* = 270, 136 female), we first explored if the same sample duration conditions used in Experiment 1 would lead to different outcomes if we reduced the parade size to include only six voices. Following this, we pooled the data from both experiments to investigate the influence of target-position effects. The results show that 15-s sample durations result in statistically equivalent voice identification performance to the longer 60-s sample durations, but that the 30-s sample duration suffers in terms of overall signal sensitivity. This pattern of results was replicated using both a nine- and a six-voice parade. Performance on target-absent parades were at chance levels in both parade sizes, and response criteria were mostly liberal. In addition, unwanted position effects were present. The results provide initial evidence that the sample duration used in a voice parade may be reduced, but we argue that the guidelines recommending a parade with nine voices should be maintained to provide additional protection for a potentially innocent suspect given the low target-absent accuracy.

## Introduction

During some crimes, the perpetrator is heard, but not seen. The severity of such crimes might vary from attempted telephone fraud to crimes such as rape or murder where disguises may be worn (*R v Khan and Bains*, 2002, discussed in Nolan, 2003). If the only identification evidence linking the perpetrator to the crime is the sound of their voice, this evidence might be decisive in court (Robson, 2017). In these situations, the person who overheard the perpetrator’s voice during the crime is often referred to as an “earwitness.” Earwitnesses may be asked by the police to try to identify the voice of a perpetrator from a voice parade.

In England and Wales, Home Office (2003) guidelines provide recommendations about how a voice parade should be constructed. The guidelines were innovative at the time of their development in that they promoted an approach to voice sample construction that was underpinned by a research-based phonetic understanding of speech. However, other aspects of the guidelines that relate to the way the procedure is conducted (i.e., how the voices are presented to witnesses) are not research-based, and, importantly, were adapted from face identification procedures^1^ rather than being designed specifically for voices (Smith et al., 2020). This article builds on the work done by Smith et al. (2020) in proposing amendments to the Home Office (2003) voice parade procedures through improved understanding of system variables (Wells, 1978), that is, variables that are controllable by law enforcement. Importantly, while the focus of this article is centred on using the guidelines developed for use in England and Wales, the conclusions of this body of research are relevant to all jurisdictions where earwitness evidence is admitted as evidence; this includes much of Europe, the United States, Australia, and Canada (Broeders & van Amelsvoort, 2001; Cantone, 2010; Laub et al., 2013; McGorrery & McMahon, 2017). The present article focuses on providing evidence-based recommendations for the system variables of *parade size* and the voice *sample duration*. The Home Office guidelines recommend that “a total of nine samples should be selected (i.e., the suspect’s plus eight others),” and that “these [samples] should each be about one minute long . . .” (Home Office, 2003, point 13). There are both practical reasons and theoretical reasons for attempting to manipulate both the number and length of the voice samples included in a voice parade, which we summarise below. We provide an overview of the current and historical limitations of earwitness research and outline how the present study attempts to overcome some of these.

### Sample duration

In England and Wales, constructing a voice parade involves several stages. First, an expert phonetician must pick out similar-sounding speakers from recorded police interviews to act as foils. These speakers are therefore suspects from different cases. The expert selects excerpts of speech from the recordings, but the content must be unrelated to the crime in question and contain no identifying information. They then splice together the speech excerpts to form voice samples for both the foils and the suspect (see Nolan, 2003, for a detailed description of the procedure forensic phoneticians use to produce the voice samples). This complicated and time-consuming process may increase the delay between the crime (encoding) and the parade (retrieval). Prolonged retention intervals risk reducing the strength of the initial memory (of overhearing the perpetrator’s voice) and make successful identification less likely (King et al., 2002). A specific practical reason for the delay in preparation is that it can be difficult to locate enough suitable recordings of voices to produce 60-s samples. Simply put, by reducing the duration of the voice samples, it would, in general, take less time to source material and to splice together the samples to be used in the parade.

While the 60-s sample duration recommendation by the Home Office seems like a reasonable amount of time needed for a listener to build a representation of a voice to compare it with their memory of the perpetrator, people can extract basic information from voices after less than a second of exposure, such as emotion (Bestelmeyer et al., 2010) and aspects of personality (McAleer et al., 2014). While unfamiliar voice identification would likely require somewhat longer exposure, it does mean that the 60-s voice sample may be unnecessarily long if it offers no advantage over shorter durations. Indeed, Broeders and van Amelsvoort (2001) suggest in their guidelines^2^ for constructing a voice parade that the voice samples need be no longer than 20 s in duration.

Empirically, the only research that has focused on addressing the effect of sample duration was conducted by Smith et al. (2020). They compared 15- and 30-s samples and found that there was no difference in accuracy, arguing that this lack of effect may have been because the amount of identity information in 15- and 30-s samples did not differ in the extent to which it activated the auditory representation of the target voice. In addition, the authors suggested that even if there was a difference in the amount of identity information between the two sample durations, the benefits were offset by the additional interference associated with longer sample durations. In line with this explanation, the interference associated with 60-s samples might even make performance worse. A limitation of the Smith et al. (2020) study, however, was that comparisons were not made against the Home Office guidelines recommending 60-s samples, meaning that the research could not be used to support procedural recommendations in England and Wales. It is important to highlight that a shorter duration does not necessarily need to result in higher accuracy for changes to be recommended; if the shorter duration is not *worse* than a longer duration, the practical utility of a shorter duration is clear.

In addition to the practical benefits of reducing the sample duration of the voices included in a voice parade, there are theoretical reasons to believe that a shorter duration may be beneficial, or at the very least not harmful to performance. First, temporal-ratio models of memory suggest that time is an important dimension which underpins memory organisation and retrieval (Bjork & Whitten, 1974; Brown et al., 2007; Crowder, 2014). The basis of temporal models of memory is that more recent items are more easily discriminable from distant items, and in order for the memory trace of later items to be distinct, a greater temporal separation is required. As the temporal gap between voices in a parade does not progressively increase, this suggests that voices with a shorter duration may be more distinct than those with longer durations, particularly for voices towards the end of the parade. Second, shorter voice samples may be beneficial for the listener due to the combination of temporal-based memory damage and interference between the voices. For instance, Kerstholt et al. (2006) suggest that following a crime, the delay between exposure and testing is typically around 3 weeks. Longer retention intervals are associated with lower voice identification accuracy (Yarmey & Matthys, 1992). In addition, Stevenage et al. (2013), Stevenage and Neil (2014) show that memory for voices is particularly vulnerable to disruption and interference. By reducing the temporal gap between exposure and testing (by reducing the resource demands of the experts constructing the voice samples) and limiting the chances for disruption that may occur in longer voice samples, earwitnesses may have a greater chance of successfully identifying the target voice as well as correctly rejecting voices that do not belong to the perpetrator.

It is helpful to frame this in terms of Signal Detection Theory (SDT; Macmillan & Creelman, 2004), which has emerged as a powerful framework for comparing the strength of witness memory accuracy using different types of identification procedures (Cohen et al., 2020). Signal detection models provide two metrics of interest. The first of these is the response criterion, which in the context of identification parades measures the participants’ willingness to respond that the signal (i.e., the target voice) is present independently of the information that the participants received. The second metric, which is of primary interest to the present article, is signal sensitivity; signal sensitivity (*d′*) is a measure of how well a listener can detect a signal among noise (DeCarlo, 1998). If the theoretical premise discussed above holds true, the increased distinctiveness of shorter samples may facilitate the ability of listeners to discriminate the signal (which would be the sample of the perpetrator’s voice in the parade) from the noise (which would be the similarly sounding foil voice samples) within a voice parade.

### Size of the parade

Just like sample duration, the size of the parade (i.e., the number of speakers in the parade) has both practical and potentially theoretical implications. The practical implications of using fewer voices are similar to that of using shorter sample durations: the resources required to set up a voice parade can all be reduced if there are fewer foils. This would ultimately lead to a shorter interval between exposure and identification, reducing the risk of memory decay.

The number of foils used in previous voice identification research has varied. Lab-based voice parades have commonly used either five (Cook & Wilding, 1997a, 1997b; Kerstholt et al., 2006; Smith & Baguley, 2014), which is in line with the recommendation put forward by Broeders and van Amelsvoort (2001), or eight (McDougall et al., 2015; Smith et al., 2020), which is what is recommended by the Home Office (2003) guidelines in England and Wales. An important benefit of a larger parade is that the additional foils offer an innocent suspect increased protection. For instance, if target-absent performance is at chance level (e.g., Sherrin, 2015), an innocent suspect has a 10% chance of being randomly selected,^3^ if they are included in a nine-voice parade compared with over a 14% chance in a six-voice parade. However, there have been few psychological studies which have experimentally tested the ideal number of foils for voice parades. One of these was a study undertaken by Bull and Clifford (1984) who compared voice parades with four, six, and eight voices and concluded that a six is the optimal number with performance dropping when parades are either longer or shorter. In contrast, a study by Hammersley and Read (1996) concluded that 20 voices is the optimal number to protect an innocent suspect. Both studies could be considered exploratory, and both had limitations: for instance, Bull and Clifford (1984) did not take into account the protection afforded to potentially innocent suspects when more foils are included, while Hammersley and Read (1996) did not take into account the resources that would be required to build 20-voice parades. Additional research is required to provide any type of evidenced-based policy recommendation regarding the number of foils used in a voice parade.

In terms of theoretical implications, listening to a series of voices in a parade requires attention. The longer and more numerous the voices, the greater the attentional resources required, and the higher the risk of cognitive overload. As Zimmermann and colleagues (2016) posit in their discussion of auditory attention and memory, the propensity for erroneous decisions is likely to be linked to high resource demand. The authors suggest that there is always a risk of erroneous processing when an incoming signal is compared with information already stored in long-term memory because this information is only an approximation of the raw input. As noted previously, comparing incoming signals (the voice samples) with information already stored in long-term memory (the perpetrator’s voice) is the essence of a voice identification parade.

In terms of SDT, this suggests that signal sensitivity strength may be negatively affected by the attentional resource requirements of the voice parade. If this holds true, reducing the number of voices, and thus attentional demands, may limit the risk of erroneous comparisons and increase signal strength. In addition to auditory attention, by reducing the total number of voices to review in the parade, there may be, as per temporal-ratio models of memory (Bjork & Whitten, 1974; Brown et al., 2007), a reduction in the optimal temporal distance between the least and most recent voices in the parade, facilitating both relative and absolute judgements.

### Confidence

Research using unfamiliar voices often shows unreliable confidence-accuracy relationships, ranging from weak or null relationships (Kerstholt et al., 2004; Öhman et al., 2011; Olsson et al., 1998; Smith et al., 2020) to significant relationships (Bull & Clifford, 1984; Saslove & Yarmey, 1980; Yarmey, 1991). Despite this unreliable relationship, self-reported confidence has been found to influence mock jurors judging the reliability of witness voice identification (Van Wallendael et al., 1994). In addition, previous research has suggested that a witness’ confidence in their judgement varies based on the perceived difficulty of the parade (Olsson, 2000). Thus, it is important to consider the potential effects of any procedural changes on listener confidence.

### Limitations of earwitness research

Apart from the dearth of research into system variables, psychological investigations into voice identification tend to exhibit limitations that the present research attempts to rectify. First, earwitness research in the psychological literature generally uses only one or two targets when designing the parades (e.g., McAllister et al., 1993; Öhman et al., 2013a; Philippon et al., 2013). This does little to alleviate concerns about whether any results are influenced by a particularly distinct target, which may affect not only the external validity of the results, but the construct validity, too (Wells & Windschitl, 1999). Research suggests that a distinctive voice is more likely to be correctly identified regardless of familiarity (Skuk & Schweinberger, 2013; Stevenage, 2018; Yarmey, 1991). This highlights the importance of the foil selection method to ensure that a fair parade is developed.

Voice parades in experimental research are often constructed relatively arbitrarily and without consideration of similarity relative to the target. While some research has used description-match and suspect-match (Tunnicliff & Clark, 2000; Wells, 1993) methods to select parade foil samples (Cook & Wilding, 1997a; Kerstholt et al., 2004), the average persons’ available vocabulary to define the characteristics of a voice is far more limited than that which can be used to define a face (see Kelly, 2018). Thus, the efficacy of these match-approaches may be limited for voice parades. A strength of the present research is the use of multidimensional scaling methods to develop quantitatively fair parades from established forensic voice databases (detailed in the online Supplementary Material A). This is the approach used by forensic phoneticians when constructing “real” voice parades (see McDougall, 2013, for more information). Thus, by adopting this approach, we maximise the ecological validity of the experiments.

Finally, the analytical procedures used to derive results and infer interpretations often do not take into account the full utility of the data. For example, Wixted et al. (2016) argues that by using alternative analytical procedures, such as SDT models as opposed to simpler regression models, we can get a more nuanced interpretation of the same data. In this study, we combine both traditional binary analyses with SDT models, which allow us to go beyond measuring accuracy and to provide metrics of decision criteria and sensitivity.

### The current study

The research presented in this article aimed to provide clarity on two system variables that are fundamental to the construction of any voice parade: the duration of the voice samples and the number of foil voices included. While the experiments undertaken were designed with the aim of testing whether the voice parade procedure outlined in the England and Wales Home Office (2003) guidelines can be improved, the outcomes are relevant to all legal jurisdictions which make use of voice parades. For instance, the guidelines presented by Broeders and van Amelsvoort (2001)—which recommend that voice samples should be 20 s in duration with a total of five foils in the voice parade—are similar to the conditions tested against the Home Office guidelines.

## Experiment 1

In Experiment 1, we investigated whether reducing the sample duration to 30 or 15 s improves witness performance (i.e., a listener’s ability to distinguish between the signal [the target] and noise [the foils]) compared with 60-s samples in a nine-voice parade. Based on temporal-ratio models of memory (Bjork & Whitten, 1974; Brown et al., 2007), we hypothesised that the reduced relative distance and chances of interference or disruption between voice samples facilitated by shorter sample duration times, as well as the reduced attentional demands, will offset any advantages of the increased amount of identity information afforded by longer sample duration times. Specifically, we hypothesised that overall accuracy rates and signal sensitivity (*d′*) would be greater when shorter sample durations are used in the voice parade. As there are no experimental manipulations specifically targeting a listener’s decision criterion (such as the instructions listeners receive before undertaking the parade; e.g., Smith et al., 2022), we do not expect meaningful differences in criterion (*c*) between the sample duration conditions.

### Method

#### Participants.^4^

A total of *N* = 277 participants were recruited via the online recruitment platform *Prolific.co*. Six participants were excluded (five for having average between-voice response times (RTs) that were clear outliers, and one due to non-completion of the filler task) making the final sample size *N* = 271. The average age of the participants was 27.68 years old (*SD* = 6.16) with 136 males and 135 females. Participants were required to (1) have been born in England, (2) have lived most of their life before turning 18 in England, (3) speak English as their first language, (4) have no uncorrected hearing loss or hearing difficulties, and (5) be between the ages of 18 and 40.^5^ In addition, participants were required to have a minimum approval rate of 90% on Prolific and a minimum internet connection speed of over 5 mb/s. Ethical approval was granted by the Schools of Business, Law and Social Sciences Research Ethics Committee, Nottingham Trent University and the Ethics Committee of the Faculty of Modern and Medieval Languages and Linguistics, University of Cambridge.

#### Design

Experiment 1 employed a between-subjects 3 (sample duration: 15, 30, or 60 s) × 2 (target: present or absent) factorial design. A Bayesian factor design analysis (Stefan et al., 2019) using small, medium, and large odds ratio effect sizes (Chen et al., 2010) indicated that, with a fixed *n* of 45 participants per condition, there is a 30.7% chance of finding support for H1 with moderate evidence (BF > 3) when the effect size is small, 86% when the effect size is medium, and 98.9% when the effect size is large. To have an 80% chance of detecting small effects with at least moderate evidence, the number of participants per condition would effectively need to be doubled. We argue that not only is this prohibitive in terms of resources, but small effects with statistical importance would not necessarily translate to practical importance, whereas larger effects—of which the current sample size provides adequate detection—would have far more practical value.

#### Apparatus and materials

The speech materials used in this study were taken from the Dynamic Variability in Speech database (*DyViS*) (Nolan et al., 2009; available to download from the UK Data Service), the York Variation in Speech database (*YorViS*) (McDougall et al., 2015; contact author for permission), and the West Yorkshire Regional English Database (*WYRED*; Gold et al., 2018, available from the UK Data Service). Further details can be found in the online Supplementary Material A, Table A1.

*DyViS*, *YorViS*, and *WYRED* each contain recordings of a relatively large number of speakers sharing the same demographic characteristics, that is, speakers of the same age (18–30 years), sex (male), and accent background (*DyViS*: Standard Southern British English (SSBE); *YorViS*: York English; *WYRED*: Bradford/Kirklees/Wakefield English), making them ideal sources of speech recordings for constructing ecologically valid voice parades. The demographic control offered by these databases enables the researcher to choose appropriate foils for comparison with a suspect as is essential in a real parade context.

All databases used the same elicitation techniques. There were two tasks relevant to the current research. One task, used for the encoding sample, was a mock telephone call between a speaker (the “perpetrator”) and an accomplice who was on the other end of the phone and whose speech was not audible. The participant who was speaking into the telephone in the studio, and being recorded at studio quality in situ, was also recorded at the remote end of the telephone line. The other task, used for the parade samples, was a simulated police interview designed to elicit spontaneous speech in a situation of cognitive conflict (i.e., lying). The speaker plays the role of a suspect being interviewed. In both tasks, the speech produced by the participant is not scripted and relatively spontaneous in style. The participant responds to questions from the accomplice (phone call) or interviewer (police interview), guided by visual information containing names of characters, streets, locations, and so on.

##### Speaker selection

To construct the parades, a total of six groups comprising 10 speakers each (1 target, 1 replacement, and 8 foils) were selected following multidimensional scaling (MDS) analysis (see the online Supplementary Material A for a detailed overview of this process). Three of these groups used SSBE speakers from the *DyViS* database, two used speakers from the *WYRED* database (one Wakefield English group, one Bradford English group), and the last featured speakers of York English from the *YorViS* database. Between-speaker variability is high (Lee et al., 2019), so stimulus sampling is crucial (Windschitl & Wells, 1996). These speaker groups were purposely selected so that variation was captured from across four accent groups (SSBE, York, Wakefield, and Bradford). Furthermore, the three SSBE groups were selected to capture variation within a single accent.^6^

##### Encoding samples

The samples of the target voice for the encoding stage of the experiment were all approximately 60 s^7^ in duration once edited and were taken from the telephone conversation with the accomplice. Participants did not hear the target’s interlocutor. These samples were used to simulate the participant (i.e., “earwitness”) overhearing a crime. Editing involved cutting pauses where the interlocutor on the other end of the phone was speaking so that the net speech was similar for all encoding samples. All clips had to be clearly audible, and hesitations that were particularly salient in terms of their length were avoided.

##### Parade samples

All the voice samples used in the parade were taken from the studio-quality simulated police interviews to replicate the process used by the police. Excerpts featuring the interviewees were spliced together to produce 15-, 30-, and 60-s samples. The voice samples were taken from different sections of each interview so that the content of speech differed across speakers.^8^ For both the encoding and parade samples, one second of silence, taken from a silent section of each respective interview, was inserted between utterances, and any extraneous noises (coughs, sneezes, chairs moving) and speech of the interlocutor were removed. Furthermore, the content of the interview samples did not overlap with the content of the phone recording. All audio stimuli were normalised for intensity. The order of the excerpts was randomised to ensure that there was no continuous narrative within each sample.

#### Procedure

##### Overview

The experiment was hosted on the online experiment builder platform Gorilla.sc (Anwyl-Irvine et al., 2020). Participants were instructed to complete the experiment in a quiet environment with no distractions. Participants were required to calibrate their headphone volume and then undertook a headphone screening assessment (Woods et al., 2017) to ensure they were using headphones or earphones. Participants were allowed two attempts to identify correctly at least four out of six of the “softest” tones—if they failed both attempts, they were rejected from the experiment.

As participants progressed through the experiment, there was no option to go back and review previous elements. Upon successful completion of the headphone screening task, the participants were randomly allocated to target-speaker groups (*DyViS* 1, 2, 3, *WYRED* 1, 2, and *YorViS*), condition (15-, 30-, 60-s samples), and type of parade (target present or target absent) using balanced randomisation. Each participant completed a single trial. They were not told that they would have to complete a voice parade, but rather that they would be undertaking an experiment on voice perception. This facilitated incidental as opposed to intentional encoding of the voice. This is important as witnesses are, arguably, more likely to be focusing on the event (incidental) rather than trying to remember the details so that they can be recalled at a later point in time (intentional) (Smith et al., 2020). In target-present parades, the target voice was either in a relatively early (third) or late (seventh) position to counterbalance for positional effects.

##### Phase 1: Encoding

Participants listened to the 60-s encoding sample when they were ready to begin the experiment by clicking a “Play” button on the screen. Participants automatically moved on to the next stage of the experiment when the encoding sample finished playing.

##### Phase 2: Storage

After listening to the encoding sample, participants completed a filler task. This task comprised a wordsearch (of different fruit names) with numbered axes. Participants were instructed to find as many words as possible and list the x- and y-axis numbers of the first letter of each word. At the same time, they listened to a recording of ambient noise, which was made in a public lobby and featured unintelligible speech sounds. A 5-min timer countdown was displayed in the top right corner of the screen. We excluded participants who entered less than five words in the filler task unless an explanation was provided in the debrief (e.g., they were not sure how to “enter in” a word).

##### Phase 3: Retrieval

Before the parade began, participants read that they would be listening to a voice parade which contained “a series of nine voices.” Participants were asked to “imagine that the voice you heard initially was one that you overheard in a public setting where you could hear but not see the speaker.” Importantly, participants were told, “the speaker may or may not be present in the parade,” and if they felt that the speaker was absent, they should indicate this by selecting the option of “none” when asked “which voice do you think belonged to the perpetrator?.” Having read the pre-parade instructions, the voice parade began immediately once participants had clicked “continue.” Each voice sample in the parade corresponded with a voice number (Voice 1, Voice 2, etc.). To encourage participants to reorient their attention prior to hearing each voice sample, they pressed the spacebar to proceed to the next voice. The recorded RTs enabled us to check that the participants were paying attention to the task (for both experiments; mean RT = 10,898 ms; *SD* = 7,319 ms). Participants were not permitted to listen to a voice again once it had finished playing, and were required to listen to all voices fully before proceeding to the testing phase.

After listening to all nine voices in the parade, participants selected which voice, if any, they thought belonged to the perpetrator. On this page, participants were reminded to select “*none*” if they thought the target speaker was absent. After registering their decision, participants rated their confidence in their decision using a 11-point slider scale, ranging from 0 (*not at all confident*) to 10 (*extremely confident*).

Participants completed a series of debrief questions after finishing the experiment. Responses to these were inspected but not formally analysed. Included in these were open-ended questions about the strategy used to identify the perpetrator, whether they considered responding that the perpetrator was not present, and if any technical difficulties were experienced. We also included a 5-point Likert-type scale asking participants to rate if they thought the parade was *very easy* (1) up to *very difficult* (5).

### Results

#### Accuracy

Data were analysed using Bayesian mixed models (Gelman et al., 2014; McElreath, 2016) with accurate parade identifications scored as 1 and inaccurate identifications as 0, in a 3 (sample duration: 15, 30, 60 s) × 2 (target presence: present or absent) factorial design.^9^ The 60-s sample duration condition was treated as the reference category. This analysis treated the six target voices as a random factor. Participants were not treated as a random factor as listeners completed a single trial. We elected to use weakly informative priors with a normal distribution N(0, 1); this is considered to be a conservative approach (Gelman & Jakulin, 2007), but one that still contains enough prior information to regularise extreme inferences obtained using completely noninformative priors (Gelman et al., 2008). In addition, when calculating Bayes factors, using weakly informative priors is a way to take into account the size of the sample and constrain the magnitude of evidence accordingly (Rosas et al., 2019). Leave-one-out cross-validation was used to evaluate model comparisons (Vehtari et al., 2017). The fitted models’ predictive performance was estimated as the sum of the expected log pointwise predictive density $\left(\hat{elpd}\right)$ alongside its standard error (*SE*). A model with a difference in *SE* (Δ*SE*) equal to or greater than 5 is suggestive of statistically better performance (Vehtari et al., 2017). While the model with only target presence included as a predictor resulted in the highest predictive performance, no model exceeded a Δ*SE* of 5. Thus, we selected the model including interactions for inference (for the full model, see the online Supplementary Material B, Table B2).

**Figure 1.**
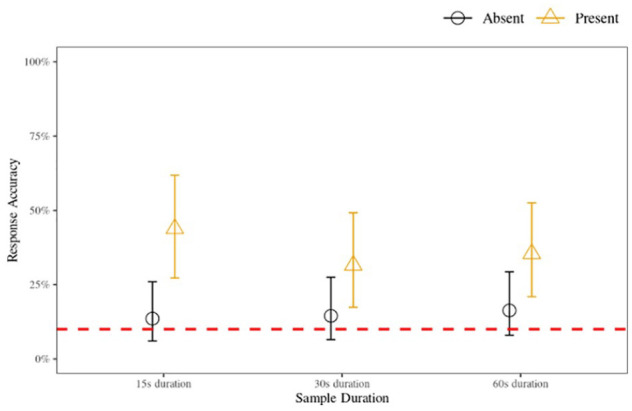
Voice identification accuracy for 15-, 30-, and 60-s sample duration conditions for target-present and target-absent parades, Experiment 1 (nine-voice parade). *Note.* The data points indicate the most likely posterior probability parameter values derived from the interaction model, and the error bars show the 95% highest density interval (HDI). The dashed line indicates chance level (10%) accuracy rates (1 / *n*_response-options_).

The results of the model indicate that there is negligible^10^ (i.e., insufficient) evidence to support the hypothesis that accuracy in the 15- and 30-s sample conditions differed meaningfully from accuracy in the 60-s sample condition. While there was moderate evidence to support the hypothesis that parades in which the target was present were more likely to be accurate compared with those where the target was absent, there was negligible evidence supporting the hypothesis that a meaningful interaction effect existed between target presence and the sample duration conditions. Overall, these results show that there is no meaningful difference in binary accuracy between the sample duration conditions. Inferring from the interactions model, the most probable parameter values (for all conditions with corresponding 95% highest density intervals [HDIs]) are illustrated in Figure 1.

#### SDT model

We evaluated voice identification in the context of equal variance Gaussian signal detection theory (EVSDT, e.g., Wixted et al., 2016). Bayesian generalised mixed-effects models (with a Bernoulli distribution) were used to infer the model parameters. Model parameters were the response criterion *c* (calculated as the negative standardised FA rate; DeCarlo, 1998), and *d′* (the difference between the standardised hit rate and the standardised FA rate) which represents listeners’ ability to detect the signal (i.e., the target voice in target-present parades, or lack of a target voice in target-absent parades) among noise (the foils). The model was implemented as non-linear syntax. We elected to use the same weakly informative priors as in the accuracy analysis, but allowed for greater variance N(0, 3). The full decision frequency for all conditions is displayed in Table 1.

**Table 1.** Decision frequency with percentages in parentheses (Experiment 1; nine-voice parade).

Sample duration	Target present (TP)			Target absent (TA)	
	Hit	Foil	Reject	Foil	Reject
15 s	20 (45%)	21 (48%)	3 (7%)	42 (87%)	6 (13%)
30 s	14 (32%)	26 (59%)	4 (9%)	40 (85%)	7 (15%)
60 s	17 (37%)	27 (59%)	2 (4%)	35 (83%)	7 (17%)
Total	51 (38%)	74 (55%)	9 (7%)	117 (85%)	20 (15%)

*Note.* TP Hits = correct target IDs; TP Foils = incorrect Foil IDs; TP Reject = incorrect rejection; TA Foils = incorrect foil IDs; TA Reject = correct rejections.

For the SDT analyses, we removed target-present foil IDs so that the parameter of interest is purely the ability of listeners to discriminate between guilty suspects and innocent suspects (e.g., Colloff et al., 2016) and not an absolute notion of discriminability (i.e., the ability to discriminate between guilty suspects, innocent suspects, and foils). As we did not include a designated innocent suspect in the target-absent parades, we applied a conventional nominal adjustment within the model syntax which adjusted the false alarm rate according to the number of voices in the parade.

The conditional *maximum a posteriori*^11^ estimates are presented in Table 2. For the parameter-value estimates, we observe that there is strong evidence supporting the hypothesis that *d′* was above zero for the 15- and 60-s duration conditions, but negligible evidence for the 30-s condition. This indicates that, on average, listeners in the 15- and 60-s, but not the 30-s, duration conditions displayed some ability to distinguish the signal from the noise. As for criterion, there is moderate evidence supporting the hypothesis that the criterion for the 15-s duration is below zero, but negligible evidence for the 30- and 60-s duration conditions. In other words, there is evidence that listeners in the 15-s, but not the 30- and 60-s, conditions adopted a liberal decision criterion. We found negligible differences when conducting pairwise comparisons for signal and criterion strength across sample durations (BF_10_ < 3).

**Table 2.** Bayesian estimates of the EVSDT model analysis for Experiment 1 (nine-voice parade). Criterion *c* represents willingness to respond target present and *d′* indicates signal sensitivity.

Condition	Sensitivity (*d′*)				Criterion (*c*)			
	$\hat{\beta }$	95% HDI	BF_01_	BF_10_	$\hat{\beta }$	95% HDI	BF_01_	BF_10_
15 s	1.15	[0.40, 2]	0.04	23.6	–0.13	[–0.21, –0.07]	0.21	4.71
30 s	0.79	[0.12, 1.61]	0.61	1.65	–0.12	[–0.20, –0.06]	0.55	1.81
60 s	1.28	[0.42, 2.25]	0.08	12.17	–0.11	[–0.19, –0.05]	0.71	1.42

*Note.* $\hat{\beta }$ = *maximum a posteriori* (MAP); 95% HDI = 95% highest density interval; BF_01_ = support for H_0_; BF_10_ = support for H_1_.

#### Confidence

Confidence ratings, on a scale of 0 (*not at all confident*) to 10 (*extremely confident*), were analysed in cumulative models for ordinal data (Bürkner & Vuorre, 2019; Liddell & Kruschke, 2018).^12^ We elected to use the same weakly informative priors as in the preceding models, allowing slight adjustments to the variance N(0, 2). We investigated the relationship between confidence ratings and accuracy for each sample duration condition separately.

**Figure 2.**
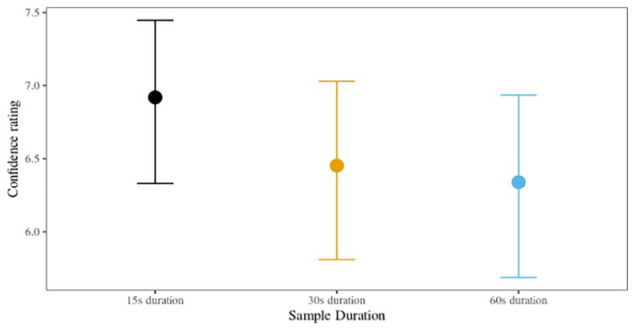
Posterior confidence with 95% HPDIs, Experiment 1 (nine-voice parade).

We found moderate evidence of a positive relationship between confidence and accuracy for the 60-s sample duration ( $\hat{\beta }$ = .91, HPDI: [0.15, 1.69], BF_10_ = 3.32). Evidence was negligible for the other sample durations (15 s: $\hat{\beta }$ = .25, HPDI: [–0.48, 0.96], BF_10_ = 0.23; 30 s: $\hat{\beta }$ = .48, HPDI: [–0.29, 1.25], BF_10_ = 0.41). In other words, participants were more confident about correct responses (than about incorrect responses) when listening to a voice parade with 60-s samples, but there was no meaningful relationship between confidence and accuracy for listeners in the 15- and 30-s conditions. Posterior cell means are shown in Figure 2 for each condition.

We also ran cumulative ordinal models to see if there were any by-sample-duration or by-target-presence differences in the perceived difficulty of the task. We found that, compared with the 60-s sample duration, there were no meaningful differences in perceived task difficulty for the 15-s ( $\hat{\beta }$ = –0.49, HPDI: [–1.2, 0.25], BF_10_ = 0.42) and 30-s ( $\hat{\beta }$ = 0.19, HPDI: [–0.53, 0.95], BF_10_ = 0.22) sample duration conditions. Likewise, there was no meaningful difference in perceived difficulty between target-absent and target-present parades ( $\hat{\beta }$ = –0.44, HPDI: [–1.14, 0.31], BF_10_ = 0.36).

### Discussion

The accuracy analysis showed that rates were low (<50%) and performance in parades where the target was absent was substantially lower than in parades where the target was present, consistent with previous findings (Kerstholt et al., 2004; Öhman et al., 2011, 2013a, 2013b; Perfect et al., 2002; Smith et al., 2020). Despite the overall low accuracy, all target-present parades had accuracy at above-chance levels. This indicates that even though the task used in the experiments was difficult—a short exposure to the target with incidental encoding, a cognitively demanding filler task, and quantitatively similar voices (i.e., a fair parade)—voice identification is difficult, but not impossible.

The signal detection analyses reveal that listeners in the 15- and 60-s duration conditions were able to distinguish the “guilty” voice from “innocent” ones. However, in the 30-s condition, listeners’ ability to distinguish guilty from innocent was no different from chance. These findings partially support our hypothesis that the increased temporal distinctiveness of the shorter 15-s sample duration conditions, as well as the reduced risk of interference and disruption, offset the benefit of the maximal amount of identity information afforded by the longer 60-s sample duration. Contrary to expectations, the results suggest that the 30-s sample duration condition does not represent the combined benefits of increased distinctiveness afforded by the shorter durations and increased identity information provided by the longer durations. Rather, the benefits of both the shorter and longer durations are reduced to a point where a compromise leads not to the “best of both worlds” but to the worst.

Contrary to expectations, we found differential effects in criterion based on the different sample durations. We found that listeners in the 15-s sample duration condition were more likely to adopt a liberal response criterion (i.e., a *c* score that was statistically meaningfully below zero) compared with listeners in the 30- and 60-s sample duration conditions, who had a neutral response criterion. This means that listeners in the 15-s sample duration condition had a heightened predisposition to respond that the target was present when undertaking a parade, regardless if the target was present or absent, translating to a higher likelihood of false alarming compared with the other duration conditions. Importantly, it is only the comparison with a completely neutral response bias (i.e., zero) that was statistically meaningful and not the magnitude of the pairwise difference of *c* between the conditions. As such, considering the substantially greater level of listener discrimination when presented with the shorter (15 s) clips than the longer (60 s) clips, as well as the reduction in resource requirement when using shorter clips, the benefits of the former appear to outweigh the risks of bias noted here.

There was a statistically meaningful relationship between confidence and accuracy in the 60-s sample duration condition, but not in the 15- or 30-s conditions. Listeners in the 60-s condition were more confident about accurate responses than inaccurate responses. The inconsistency of the results is consistent with the largely tenuous confidence–accuracy relationships reported in other earwitness research (Kerstholt et al., 2004; Öhman et al., 2011; Perfect et al., 2002). It is possible that longer exposure to the voice information in this sample condition, in conjunction with meaningful signal sensitivity, facilitated this confidence–accuracy relationship in the 60-s condition, but not the shorter sample duration conditions. The analysis of perceived task difficulty indicated that no experimental condition was perceived as being statistically easier or more difficult than the others, suggesting that decision confidence was unrelated to the difficulty of the task.

Overall, the results of this experiment provide initial evidence that shorter voice durations may be a feasible procedural change to the longer sample durations currently recommended in the Home Office (2003) guidelines. While further research is required to support this finding, we argue that the robust experimental design used in the present research, as well as the focus on creating ecologically valid voice parades using the expertise of forensic phonetic experts, is highly suggestive of a valid and reliable outcome.

## Experiment 2

In Experiment 2, we investigated whether reducing the number of foils in a voice parade from eight to five would change the pattern of results found in Experiment 1. This question has international relevance. While the Home Office recommends nine-person parades in line with face identification procedures, in the Netherlands, Broeders and van Amelsvoort (2001) advocate six-voice parades. Face identification procedures in the United States are also based on there being six members (e.g., see Seale-Carlisle et al., 2019). We hypothesised that due to the reduced number of voices, the auditory attentional demands (Zimmermann et al., 2016) would be reduced. This reduction in cognitive demand would result in fewer erroneous comparisons and subsequently facilitate a stronger signal sensitivity. In other words, we hypothesised that listeners undertaking a six-voice, as opposed to a nine-voice parade, would display stronger *d′* scores, as well as improved overall binary accuracy. However, based on the results of Experiment 1, we did not expect any statistically meaningful by-duration differences in overall accuracy to be found. That being said, based on the results of the first experiment, we predicted that the magnitude of signal sensitivity (*d′*) and response criterion (*c*) may differ between the sample duration conditions. We explored whether binary accuracy varied between the various target groups which were treated as random effects in the various models. As the experimental design included target speakers in both early and late positions, and previous eyewitness research has found “unwanted position effects” in sequential parades (Meisters et al., 2018), we also investigated if such position effects were present in the current data. Thus, following the primary analyses, we pooled the data from both experiments and collapsed the sample duration conditions to allow for a comparison between the two parade sizes. This included an analysis of by-parade-size accuracy, SDT models, whether the position of the target affected witness performance, and whether this differed between nine- and six-voice parades.

### Method

Apart from the following exceptions, the materials and methods were identical to Experiment 1.

#### Participants

A total of *N* = 278 participants were recruited using *Prolific.co*. None had taken part in Experiment 1. A total of eight participants had their data discarded (six for exceeding the between-voice RT manipulation check and two due to non-completion of the filler task), making the final sample size *N* = 270. The average age of the participants was 28.82 years (*SD* = 6.27), with 134 males and 136 females.

#### Procedure

The parades contained six voices (one target, five foils in the target-present parade; six foils in the target-absent parade). To account for the reduced overall parade size, the target positions changed from Positions 3 and 7 in the nine-voice parade to Positions 2 and 5 (Carlson et al., 2008; Smith & Baguley, 2014).

Whereas in Experiment 1 we constructed parades using the 10 speakers assessed by listener participants as most similar-sounding to each other within each original group of 15 (MDS analysis; see Supplementary Material A), in Experiment 2, we constructed parades using the seven speakers that listeners assessed as most similar-sounding to each other using the same set of similarity judgements, that is, we effectively dropped from each parade the three speakers with the highest Euclidean distances from the target. The target speakers and replacement foils were the same in both experiments.

### Results

#### Accuracy

We used the same analytic procedure here that was used in Experiment 1. Accurate parade identifications were scored as 1 and inaccurate identifications as 0, in a 3 (sample duration: 15, 30, 60 s) × 2 (target presence: present or absent) factorial design. This analysis treated the parade target (a total of six different parades and corresponding targets) as a random factor. Model predictors and their interaction terms were added incrementally to the intercept-only model and compared using cross-validation techniques. The fitted models’ predictive performance was estimated as the sum of the expected log pointwise predictive density (*elpd*) alongside its *SE*. A model with a Δ*SE* equal to or greater than 5 is suggestive of better predictive performance (Vehtari et al., 2017). As in Experiment 1, while the model with only target presence included as a predictor resulted in the highest predictive performance, no model exceeded a Δ*SE* of 5 (see Supplementary Material C, Table C1 for the full model comparison). For this reason, we selected the model which included the interaction effects.

**Figure 3.**
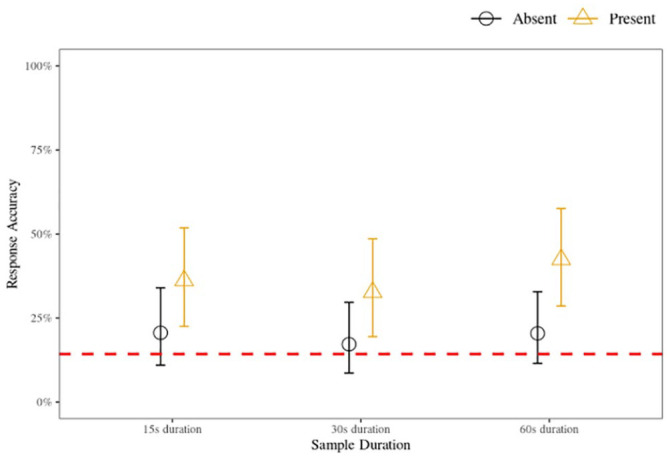
Voice identification accuracy for 15-, 30-, and 60-s sample duration conditions for target-present and target-absent parades, Experiment 2 (six-voice parade). *Note.* The data points indicate the most likely posterior probability parameter values derived from the interaction model, and the error bars show the 95% highest density interval (HDI). The dashed line indicates chance level (14.2%; 1 / *n*_response-options_).

The model, shown in full in the Supplementary Material C, Table C2, illustrates that there is negligible (i.e., insufficient) evidence to support the hypothesis that the 15-s or the 30-s sample durations differed in a statistically meaningful way from the reference category of 60-s sample duration condition. While there was strong evidence to support the hypothesis that responses to parades in which the target was present were more likely to be accurate compared with those where the target was absent, there was negligible evidence supporting the hypothesis that a meaningful interaction effect existed between target presence and sample duration. Inferring from the interaction model, the most probable parameter values (*u*) for all conditions with corresponding 95% HDIs are illustrated in Figure 3.

#### SDT model

We used the same modelling approach as in Experiment 1. We evaluated voice identification in the context of EVSDT (e.g., Wixted et al., 2016). Model parameters were the response criterion *c* and *d′.* The model was implemented as non-linear syntax. The only difference was that the nominal parade size adjustment was for six voices and not nine voices. Again, we removed target-present foil IDs from the analysis to measure discrimination only between guilty and innocent suspects (e.g., Colloff et al., 2016). See Table 3 for the complete decision frequencies.

**Table 3.** Decision frequency with percentages in parentheses (Experiment 2, six-voice parade).

Sample duration	Target present (TP)			Target absent (TA)	
	Hit	Foil	Reject	Foil	Reject
15 s	16 (36%)	26 (58%)	3 (7%)	36 (78%)	10 (22%)
30 s	14 (33%)	22 (51%)	7 (16%)	37 (82%)	8 (18%)
60 s	21 (46%)	21 (46%)	4 (9%)	37 (82%)	8 (18%)
Total	51 (38%)	69 (51%)	14 (10%)	110 (81%)	26 (19%)

*Note.* TP Hits = correct target IDs; TP Foils = incorrect Foil IDs; TP Reject = incorrect rejection; TA Foils = incorrect foil IDs; TA Reject = correct rejections.

The conditional *maximum a posteriori* estimates are presented in Table 4. As in Experiment 1, there was moderate to strong evidence in support of the hypothesis that signal sensitivity was significantly above zero in the 15- and 60-s duration conditions, but negligible evidence that signal sensitivity differed from zero in the 30-s duration condition. We observed moderate evidence in support of the hypothesis that the response criterion is lower than zero for the 30- and 60-s duration conditions, but negligible evidence for the 15-s duration condition. This suggests that, in contrast to Experiment 1, listeners in the 30- and 60-s, but not the 15-s, duration conditions adopted a liberal decision criterion for all sample durations. Pairwise comparisons revealed no reliable differences for criterion or sensitivity (BF_10_ < 3).

**Table 4.** Bayesian estimates of the EVSDT model analysis for Experiment 2 (six-voice parade). Criterion *c* represents willingness to respond target present and *d′* indicates signal sensitivity.

Condition	Sensitivity (*d′*)				Criterion (c)			
	$\hat{\beta }$	95% HDI	BF_01_	BF_10_	$\hat{\beta }$	95% HDI	BF_01_	BF_10_
15 s	1.42	[0.42, 2.68]	0.06	16.62	–0.14	[–0.22, –0.06]	0.39	2.56
30 s	0.71	[0.05, 1.77]	1.85	0.53	–0.16	[–0.24, –0.08]	0.18	5.33
60 s	1.34	[0.40, 2.53]	0.1	9.75	–0.16	[–0.24, –0.08]	0.12	8.07

*Note.* $\hat{\beta }$ = *maximum a posteriori* (MAP); 95% HDI = 95% highest density interval; BF_01_ = support for H_0_; BF_10_ = support for H_1_.

#### Confidence

Confidence ratings, on a scale of 0 (*not at all confident*) to 10 (*extremely confident*), were analysed in cumulative models for ordinal data (Bürkner & Vuorre, 2019; Liddell & Kruschke, 2018). We investigated the relationship between confidence ratings and accuracy for each sample duration condition separately.

**Figure 4.**
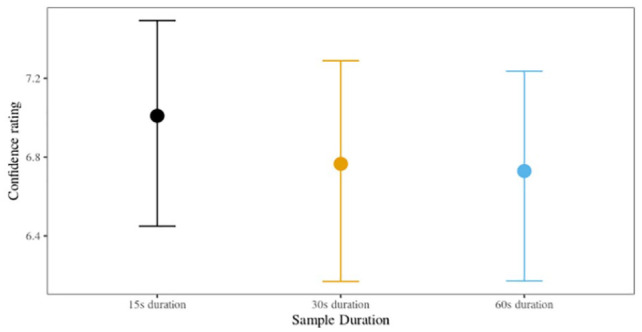
Posterior confidence with 95% HPDIs, Experiment 2 (six-voice parade).

We found moderate evidence of a positive relationship between confidence and accuracy for the 15-s sample duration condition ( $\hat{\beta }$ = .9, HPDI: [0.2, 1.66], BF_10_ = 4.2). Evidence was negligible for all other sample durations (30 s: $\hat{\beta }$ = .71, HPDI: [–0.05, 1.5], BF_10_ = 0.23; 60 s: $\hat{\beta }$ = .48, HPDI: [–0.54, 0.93], BF_10_ = 0.21). In other words, participants were more confident about correct responses (than about incorrect responses) when listening to a voice parade with 15-s samples, but there was no meaningful relationship between confidence and accuracy for listeners in the 30- and 60-s duration conditions. Posterior cell means are shown in Figure 4 for each condition.

#### Pooled Analysis

As only nominal differences in overall accuracy were found between the sample duration conditions in either experiment, we collapsed the data across parade size and investigated if any statistically meaningful differences in sample duration would emerge with the larger sample size. In addition, this allowed us to investigate by-parade-size differences using both simple accuracy and SDT metrics. Mirroring the analyses in Experiments 1 and 2, we found insufficient evidence to suggest that by-sample-duration differences were present. In addition, there was insufficient evidence to support the hypothesis that overall accuracy differed meaningfully between a nine- and six-voice parade size, nor were there any statistically meaningful two-way interactions; the only statistically meaningful predictor of accuracy was whether the parades contained a target or not. See Supplementary Material D for the full results.

We then ran an SDT model focusing on by-parade-size differences in *c* and *d′*, adjusting the FA for each parade size, respectively. The results show strong evidence that listeners in the six-voice parade displayed a liberal response criterion (*c* = –0.15, HPDI: [–0.21, –0.09], BF_10_ = 13.38) and moderate evidence of signal sensitivity (*d′* = 0.85, HPDI: [0.27, 1.44], BF_10_ = 7.59). Listeners in the nine-voice parade displayed a neutral response criterion (*c* = –0.12, HPDI: [–0.17, –0.07], BF_10_ = 2.21) and strong evidence of signal sensitivity (*d′* = 1.03, HPDI: [0.41, 1.63], BF_10_ = 26.78). There was no evidence of pairwise differences between the two parade size conditions. Overall, these results indicate that there is a greater predisposition to respond “present” in six-voice parades compared with nine-voice parades, but that both parade size conditions display meaningful evidence of listeners being able to distinguish a “target” from an “innocent,” although the evidence is substantially stronger in a nine-voice parade.

Following this, we explored whether parade-target variation existed for overall accuracy. While there was nominal variation in median accuracy between the six different speaker groups, there was no evidence to support the hypothesis that these differences were statistically meaningful (see Supplementary Material D for the parameter-value estimates and coinciding 95% HDIs). See Supplementary Material D, Figure D1, for an illustration of the cell means.

**Figure 5.**
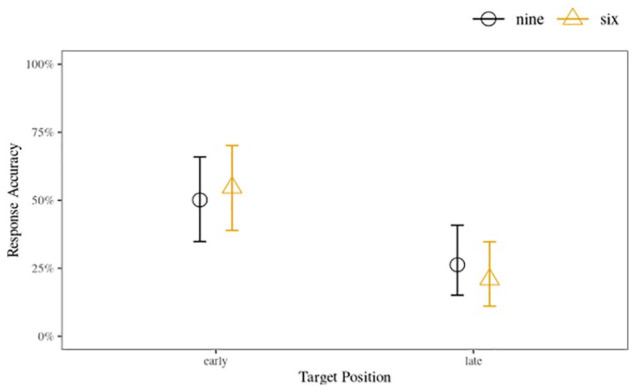
Early and late position posterior accuracy with 95% HDIs between parade size conditions, combined data from Experiments 1 (nine voices) and 2 (six voices).

Next, we investigated if positional effects played affected accuracy in target-present parades. Recall that both the nine- and six-voice parades had an “early” voice position, Voices 3 and 2 for the respective parade sizes, and a “late” voice position, corresponding with Voices 7 and 5, respectively. We analysed these data using a similar approach to the accuracy analyses: we treated TP accuracy as a binary outcome (0 = accurate; 1 = incorrect), and included the predictors of position, parade size, and their interactions iteratively. The different target voices (i.e., six different speaker groups) were treated as a random factor. We found strong evidence supporting the hypothesis that parades which had targets in the later positions resulted in lower accuracy compared with parades which had targets in the earlier positions ( $\hat{\beta }$ = –1.04, 95% HDI: [–1.70, –0.4], BF_10_ = 55.58). There was insufficient evidence to suggest that accuracy differed between parade sizes ( $\hat{\beta }$ = 0.20, 95% HDI: [–0.45, 0.8], BF_10_ = 0.372, BF_01_ = 2.63), nor was there sufficient evidence of an interaction between target position and parade size ( $\hat{\beta }$ = –0.39, 95% HDI: [–1.39, 0.42], BF_10_ = 0.79, BF_01_ = 1.26), suggesting that the positional effects were present and consistent in both parade sizes. These results are illustrated in Figure 5.

Finally, we looked at whether the perceived difficulty of the parade differs based on whether six or nine voices are used, collapsing across sample duration and target presence. On average, listeners in the six-voice parade (*û* = 3.36) perceived the task to be slightly easier than listeners in the nine-voice parade (*û* = 3.64) $\hat{\beta }$ = –0.5, 95% HDI: [–0.81, –0.2], BF_10_ = 14.97).

### Discussion

As in the first experiment, the traditional binary analysis showed that accuracy rates were low (<50%) and performance in parades where the target was absent was substantially lower than in parades where the target was present. All target-present parades, but no target-absent parades, had accuracy at above-chance levels. As expected, there were no statistically meaningful by-duration differences in accuracy. Combining the data of both experiments, the pattern of outcomes was unchanged despite the increased sample size and subsequent precision of the parameter estimates. This suggests that regardless of whether there were six or nine voices in the parade, there are unlikely to be differences in accuracy across 15-, 30-, or 60-s parade sample durations.

The pattern of sensitivity results found in the first experiment was replicated in the second experiment; that is, *d′* values were meaningfully above zero for the 15- and 60-s conditions, but not for the 30-s condition. This reinforces the previous conclusion that the 30-s sample duration is not a suitable compromise between the shorter (15 s) and longer (60 s) duration samples. In partial alignment with our hypothesis, we found that the overall strength of signal sensitivity was higher for the 15- and 60-s conditions in the six-voice parade compared with the nine-voice parade, while the 30-s condition had a reduced strength. However, the by-parade-size differences between sensitivity scores are unlikely to be statistically meaningful given the overlapping intervals. Indeed, when directly comparing six- and nine-voice parades, both displayed evidence of listeners being able to distinguish the target voice from the foils with no meaningful pairwise differences. This suggests that the benefits of reduced auditory attentional demands (Zimmermann et al., 2016) provided by a parade with fewer voices are negligible. Importantly, however, direct comparisons of the two parade sizes showed evidence that listeners display a meaningful predisposition to respond “present” in the six-voice parade, but not the nine-voice parade. This has implications for the chances of an innocent suspect being incorrectly selected and is discussed in more detail in the “General Discussion.”

We found that listeners in the 30- and 60-s sample duration conditions were more likely to adopt a liberal response criterion. However, listeners in the 15-s sample duration condition displayed a neutral response criterion. This contrasts with Experiment 1 where only listeners in the 15-s duration condition displayed a liberal response criterion. Also standing in contrast with the nine-voice parade results, we found that listeners in the 15-s condition, but not the other sample durations, were more confident about accurate responses than inaccurate ones. As in the first experiment, there were no statistically meaningful differences in the perceived difficulty of the six-voice parade conditions, suggesting that self-rated confidence was unrelated to task difficulty. Overall, listeners perceived that the six-voice parade was slightly easier than the nine-voice parade, which may reflect the reduced cognitive load required when reducing the number of foils.

Importantly, we found strong evidence that “unwanted position effects” were present in both experiments indicating that listeners in both the nine- and six-voice parades were substantially more likely to identify the target correctly when it appeared in an early position compared with a late position. While position effects have not been studied previously in voice identification research, there is some evidence that listeners are more likely to pick early faces in sequential eyewitness lineups (e.g., Nyman et al., 2020). We discuss the potential implications of these findings below.

## General discussion

We focused on the effect of two procedural manipulations on voice identification accuracy. Specifically, we tested how different sample durations (Experiment 1) and different parade sizes (Experiment 2) affect accuracy, signal detection measures, and self-rated confidence. The design of the experiment was motivated in a large part by the voice parade construction guidelines put forward by the Home Office (2003) in England and Wales but has implications which extend directly to other judicial areas, such as those put forward by Broeders and van Amelsvoort (2001). For instance, the Home Office guidelines suggest that samples in the voice parade should be at least 60 s in duration, while Broeders and van Amelsvoort (2001) suggest that the duration should be no more than 20 s. In addition, the Home Office guidelines suggest that voice parades should have eight foils, while Broeders and van Amelsvoort (2001) suggest that five foils is sufficient. Both sets of guidelines (and likely any other guidelines that have been established) were heavily influenced by existing eyewitness procedures and thus require empirical testing to ensure that they recommend parameters that are optimal for the purpose of voice identification.

We argued that shorter sample durations and fewer voices in a parade would make voice parade construction less resource intensive, thereby reducing a potential delay—and subsequent memory decay—between initial exposure to a perpetrator’s voice and subsequent voice identification procedure. We proposed that the reduced identity information afforded to listeners by shorter voice sample durations would be offset by the increased discriminability of each of the voices, as per temporal-ratio models of memory (Bjork & Whitten, 1974; Brown et al., 2007; Crowder, 2014) and reduced chances of interference between the voices (Stevenage et al., 2013, Stevenage & Neil, 2014). We also proposed that having fewer voices in a parade would have a positive effect on identification performance due to reduced attentional resources being required and a reduced risk of erroneous comparisons between the voice parade samples and the target voice (Zimmermann et al., 2016).

The results partially supported our hypotheses. In Experiment 1, we found that the performance on parades made up of 15-s samples was largely on par with performance on parades made up of 60-s samples, but signal sensitivity was at chance levels for the 30-s condition. The pattern of results found using a nine-voice parade in Experiment 1 was largely replicated using a six-voice parade in Experiment 2, but, as hypothesised, there were by-sample duration differences in the magnitude of the effects. Specifically, signal sensitivity was marginally stronger for the 15- and 60-s conditions. While we did not predict that response criterion would be influenced by either sample duration or parade size, we found evidence suggesting a tendency to respond “present” in the 15-s condition in Experiment 1 only, while in Experiment 2, the 30- and 60-s conditions, but not the 15-s condition, showed this tendency.

The results of the accuracy analyses for both experiments underline the error-prone nature of voice identification. Consistent with previous research on voice identification, our results reveal low overall accuracy and particularly high error-rates when the target is not present (e.g., Kerstholt et al., 2004, 2006; Öhman et al., 2011, 2013a, 2013b; Perfect et al., 2002; Smith et al., 2020). We found that, in terms of accuracy, the shorter sample durations (i.e., 15 and 30 s) are neither better nor worse than the Home Office recommended 60-s duration. In both experiments, the results of the SDT analysis paint a slightly more nuanced picture, highlighting the value of moving away from a purely binary analytical approach. The signal detection analysis revealed that sensitivity—which in the present research represented the ability of listeners to discriminate between guilty suspects and innocent suspects (Colloff et al., 2016)—is significantly above chance in the shortest and longest, but not the middle-most sample durations. In terms of maximising listener signal sensitivity between a guilty and innocent voice sample, out of the three durations tested, the most effective sample duration to use in a voice parade would either be 15 or 60 s. This suggests that there is a benefit of using shorter samples and a benefit of using longer samples. Shorter samples likely have the advantage of reducing competing information and memory demands. In addition, as per temporal-ratio models of memory (Brown et al., 2007), shorter samples may also be more distinct. On the contrary, longer samples likely have the advantage of enabling the listener to build a stronger identity representation. At 30 s, the identity representation might not be sufficiently strong to offset the disadvantage associated with memory demands, so there is no middle ground “sweet spot.” It is worth mentioning that this pattern of results is unlikely to be accounted for by the content or nature of the samples, which were all constructed in the same way from simulated police interview recordings. Separate utterances from the interviewee were spliced together, with 1-s silences inserted between each utterance. There was no discernible narrative in any of the samples as the order of excerpts within each sample was randomised. Therefore, the identity information in the longer samples is likely to be quantitatively rather than qualitatively different; it is not the case that speakers in the latter part of the 60-s sample have got into a speaking stride. If both short and long voice samples have similar identification performance outcomes, then it seems practical in terms of resource requirements for parade construction, as well as the subsequent facilitation of a shorter retention interval, that the shorter option should be advocated pending further empirical replications.

We predicted that a parade with fewer voices would result in improved identification performance compared with a parade with more voices based on the idea that the parade with fewer voices would free up cognitive demands relating to auditory attention (Zimmermann et al., 2016), allowing greater resources to be given to the fewer voices and reducing erroneous comparisons. As predicted based on the results of the nine-voice parade experiment, there were marginal increases in sensitivity strength for the 15- and 60-s durations when participants responded to a six-voice parade. However, when pooling the data from both experiments, there was negligible evidence suggesting that fewer voices in the parade resulted in a statistically meaningful improvement in performance across any condition. This indicates that auditory attentional requirements to generate and compare identity-percepts from voices do not differ substantially between the two parade sizes.

A more fine-grained inspection of the target-position accuracy rates between early and late positions in both six- and nine-voice parades offers a possible explanation for the negligible effect of reducing the size of the parade. The analysis showed that target voices in earlier positions resulted in accuracy rates that were almost double the accuracy for later positions. This indicates that serial voice parades suffer from the same “unwanted position effects” found in sequential eyewitness lineups (Meisters et al., 2018; Nyman et al., 2020). One explanation for this is that more auditory attentional resources are available in the beginning of a voice parade compared with voices later in the parade. This increased attentional availability facilitates more robust identity-relevant information capture for these early positioned voices, while voices later in the parade receive less attention and subsequently weaker identity-percepts, possibly due to attentional fatigue and a corresponding decrease in task motivation (Moore et al., 2017) or even interference between the voices (Stevenage and Neil 2014). The fact that both the nine- and six-voice parades showed remarkably similar position effects would support this explanation. The randomisation of voice samples, including the target position, within the parade as recommended in the guidelines produced by Broeders and van Amelsvoort (2001) may help to alleviate such positional effects.

The relatively similar performance in accuracy between six- and nine-voice parades might initially lead one to conclude that the six-voice parade should be recommended because it would reduce the amount of resources required to construct and administer the parade at no cost to identification performance. Indeed, we derive our suggestions that shorter voice samples could be adopted using this same null-effect logic. However, in an applied setting, it is vital that the inherent risks must be balanced against each other. These risks have different weights. Thus, we argue that the increased protection afforded to innocent suspects in the Home Office (2003) recommended nine-voice parade, rather than the six-voice parade recommended by Broeders and van Amelsvoort (2001), supersedes any benefits of reduced resource requirements provided by the smaller parade. To elaborate: target-absent parades simulate an innocent suspect having been apprehended, so if performance on target-absent parades is at chance level, there is a greater likelihood of an innocent suspect being randomly selected in a six-voice parade (14.2% chance of randomly selecting the innocent) compared with a nine-voice parade (10% chance of randomly selecting the innocent). When pooling the data and collapsing across the different sample durations, we found that six-voice parades were associated with a statistically meaningful liberal response criterion, while the nine-voice parade was not. In addition, while both parade sizes showed above-chance levels of signal sensitivity, the magnitude of *d′* and overall supporting evidence was stronger in the nine-voice parade. Taking these findings into account, and considering that identification research in general has shown a robust “proclivity to choose” effect (Baldassari et al., 2019) and that identification when the target is not present is notoriously error prone (Kerstholt et al., 2004, 2006; Öhman et al., 2011, 2013a, 2013b; Perfect et al., 2002; Smith et al., 2020), we feel that the risks of a parade with fewer voices outweigh the benefits of the reduced resource requirements. The fact that unreliable earwitness voice identification has contributed to wrongful convictions (Sherrin, 2015) further supports taking a cautionary approach.

It is important to highlight here that while target-absent accuracy was at chance level, target-present accuracy was consistently above chance across both experiments; that is, for all sample duration conditions and both parade sizes. The voice parades used in this experiment are very difficult, but still not impossible. Based on the related notions of propitious heterogeneity (Wells, 1993) and filler syphoning (Wells et al., 2015), the similarity of the voices used in the present experiment is likely to make accurate identifications of a target voice particularly challenging even if the listener formed a robust memory of the voices. Indeed, the accuracy rates reported in the present study might be lower than those occurring in a “real-world” situation. For instance, we would expect that the witness had been exposed to the perpetrator’s voice for longer than 1 min^13^ (as was the encoding duration used for both experiments), and it is feasible that the witness might have had some level of preparation to undertake the parade (i.e., rehearsing what they had heard) and subsequent intention to recall the voice heard at the crime scene (as opposed to the current experiment being designed specifically to minimise rehearsal and reflection of the exposure). In addition, the voices used as foils were drawn from a forensic database which matched age, gender, and location before being quantitatively assessed for similarity with the target voice. It is unlikely that any “real” voice parades would have such a homogeneous selection of voice samples to choose from.

In terms of self-rated confidence in earwitness decisions, levels tended to fall within the middle of the scale used which might reflect the uncertainty that listeners felt about their decision. The relationship between confidence and accuracy differed between the two experiments: in the nine-voice parade, there was a positive association between confidence and accuracy for the 60-s sample duration, and in the six-voice parade, there was a positive association for the 15-s sample duration. It is possible that the different confidence-accuracy outcomes in the two experiments may have been influenced by the listeners’ perception of the task difficulty. Listeners in the six-voice parade rated the task as being slightly (but statistically significantly) easier than the nine-voice parade. Thus, because the nine-voice parade was viewed as being more difficult, the listeners may have made more accurate confidence assessments of their decisions when exposed to greater identity information in the 60-s sample duration condition, compared with the shorter durations. On the other hand, when the task was rated as being easier, shorter sample durations with less possibilities of interference and reduced attentional demands may have resulted in confidence ratings that were more diagnostic of accuracy. To our knowledge, no other voice identification research has investigated perceptions of difficulty, making comparisons impossible. However, because confidence-accuracy relationships in unfamiliar voice identification have been shown to be largely unreliable weak or null relationships (Kerstholt et al., 2004; Öhman et al., 2011; Olsson et al., 1998; Smith et al., 2020; see Yarmey, 1991, for an exception), we would caution reliance on confidence as a diagnostic of accuracy until we understand more about the variables that affect this relationship.

Developing and improving voice parade procedures demands an interdisciplinary approach. To the best of our knowledge, no previous psychology studies have taken such an approach. It is often experts in disciplines other than psychology, such as phonetics and linguistics, who are asked to assist with the preparation of voice parade samples in real cases. To determine the most effective way to conduct a voice parade, expertise and knowledge from a range of disciplines need to be drawn on in concert. Relevant topics include speech behaviour and speaker variation, psychological research on memory and witness behaviour, and input from criminology concerning police produce and interaction. The present research has benefitted from such an approach, from conceptualisation and design to interpretation. The benefits are particularly apparent in terms of ecologically valid parade construction and voice sample selection. Furthermore, the six speaker groups sample variation both between- and within-accent groups, to improve generalisability in contrast to much of the previous earwitness literature which only tests identification performance in response to one or two targets (e.g., McAllister et al., 1993; Öhman et al., 2013a; Philippon et al., 2013). We feel strongly that the research presented in this article offers a procedural and methodological framework for undertaking evidence-based approaches to improving voice identification procedures.

Undoubtedly, one of the major focal points of future voice identification parade research needs to be on improving the false alarm rate while maintaining or simultaneously increasing the hit rate. The high choosing rates evidenced by the statistically meaningful liberal criterion value and a general inability to identify that the perpetrator’s voice is not present at levels that are above chance, compounded with the fact that target-absent parades are simulations of an innocent suspect scenario, paints a picture which highlights the urgent need for voice identification procedures to be updated.

Despite our attempts to overcome many of the weaknesses prevalent in voice identification research, the experiments we present still have some limitations. However, we are confident that these limitations do not undermine the conclusions we draw. First, the sample sizes for both experiments were determined by the general norms in the literature (i.e., what Lakens, 2022, calls heuristic justification) and the resource constraints (i.e., the cost). While the sample size used for both experiments was either on par with, or exceeded, those used in many previous voice identification experiments (e.g., Kerstholt et al., 2006; Perfect et al., 2002; Philippon et al., 2013; Smith et al., 2020), in some cases, there was insufficient evidence to support either the null or alternative hypotheses. While future research should strive for sample sizes large enough to provide non-negligible evidence for small effects, we have provided evidence that any effect with sufficient strength and reliability to prompt a recommendation of procedural changes had a strong chance of being observed with the current sample size.

## Conclusion

The current article adds to the slowly growing literature which highlights the value of system variable research in voice identification. The experiments undertaken were novel as they directly compared UK Home Office guidelines for constructing voice parades. Despite the regional focus of the design, the implications of the resulting outcomes have the potential to contribute to voice parade guidelines at a much larger scale where empirical evidence is often sorely lacking. Importantly, the development of the parades and the selection and editing of the voice samples, from a psychological perspective, are novel due to the strong interdisciplinary approach that was adopted and implemented in all stages. Through this collaboration, we have outlined a method of parade construction and voice selection that is both rigorous and replicable, and that maximises ecological validity. We provide initial evidence indicating that the longer voice sample duration recommended by the Home Office to be used in voice parades may be reduced without decreasing successful identification rates, while potentially saving time and other police resources. We also demonstrate that there is currently no empirical justification to support the idea that parade size be reduced from the recommended nine voices, at least until further research identifies how target-absent false alarm rates can be substantially and reliably reduced.

## Supplemental Material

sj-docx-1-qjp-10.1177_17470218231155738 – Supplemental material for Identifying unfamiliar voices: Examining the system variables of sample duration and parade size Click here for additional data file. Supplemental material, sj-docx-1-qjp-10.1177_17470218231155738 for Identifying unfamiliar voices: Examining the system variables of sample duration and parade size by Nikolas Pautz, Kirsty McDougall, Katrin Mueller-Johnson, Francis Nolan, Alice Paver and Harriet M. J. Smith in Quarterly Journal of Experimental Psychology

## Notes

1. Professor Francis Nolan notes that at the time the police were in favour of making the procedural aspects as close as possible to the visual procedure to minimise the opportunity for challenge by the defence.

2. These guidelines are partly based on the method developed by the Dutch police and judiciary for eyewitness identification (Broeders & van Amelsvoort, 2001).

3. An earwitness undertaking a parade with nine voices will have 10 possible options to choose from—one of the nine voices or none of the voices—but they can only select one of these options. If a listener were to randomly select an option, they would have a 10% chance of selecting the suspect if the suspect is included as well as a 10% chance of correctly selecting that the target was not present in a target-absent condition.

4. The sample size was justified by balancing heuristic norms and resources constraints (Lakens, 2022). In terms of the heuristic norms, the sample size used here is either on par with, or exceeds, those used in previous voice identification research (e.g., Kerstholt et al., 2006; Perfect et al., 2002; Philippon et al., 2013; Smith et al., 2020). The present research was also constrained by the cost of participant recruitment. Unlike eyewitness identification research which allows for the possibility of each participant completing multiple parades and therefore providing multiple data points (Mansour et al., 2017), earwitness identification research is often limited to a single data point per participant (largely due to interference negatively affecting auditory memory for voices, see Stevenage et al., 2011).

5. This eligibility criterion was implemented to minimise the chance of participants having age-related hearing decay (Hoffman et al., 2017).

6. While we undertake a simple analysis of target-speaker variation in accuracy, an analysis focusing specifically on accent is beyond the scope of this article. In a parallel project, we thoroughly investigate accent effects in voice identification, see for example, McDougall (2021).

7. A 60-s encoding sample is somewhat longer than the samples in some other voice identification studies (e.g., Smith et al., 2020). As some previous studies suggest that performance is likely to be better when encoding samples are longer (e.g., Legge et al., 1984; Orchard & Yarmey, 1995 but see Kerstholt et al., 2004), we hoped to avoid low performance masking the magnitude of effects.

8. It is not possible for the content of the recordings to be the same across parade members as, in the same way as with a real parade, the excerpts are taken from a recording of spontaneous speech. However, the greatest effort was taken to ensure that the content of the excerpts was as innocuous as possible, containing no information specifically relating to the fictional crime being discussed in the original recordings.

9. While there are alternative analytical approaches to investigating identification accuracy (e.g., Rosas et al., 2019; Yarmey et al., 1994), we have selected approaches that facilitate comparison with the extant literature (e.g., Kerstholt et al., 2006; Smith et al., 2020) while also incorporating analytical techniques that have been widely adopted in the eyewitness literature (e.g., Colloff et al., 2016) and are being incorporated in the earwitness literature (e.g., Smith et al., 2022).

10. Negligible here refers to a Bayes Factor in support of the alternative hypothesis (BF_10_) being less than 3; while a BF_01_ greater than 3 can technically be interpreted as evidence in support of the null hypothesis, we argue that accepting the null hypothesis requires substantially more data points per cell.

11. For all inferences, we present the *maximum a posteriori*, which is a point estimate of an unknown quantity that is equivalent to the mode of the posterior distribution.

12. While it is not uncommon for ordinal data to be analysed using methods that assume metric responses, this practice can lead to errors in inference (Liddell & Kruschke, 2018).

13. This is because forensic experts would generally not consider asking a witness to undertake a voice parade if the witness was only exposed to the perpetrator’s voice for a brief period of time.
