# Supplementary material for: Identifying unfamiliar voices: Examining the system variables of sample duration and parade size
Source: Q J Exp Psychol (Hove). 2023 Mar 7;76(12):2804–22. doi: 10.1177/17470218231155738 (PMC10655699; doi:10.1177/17470218231155738)
Supplement: sj-docx-1-qjp-10.1177_17470218231155738 – Supplemental material for Identifying unfamiliar voices: Examining the system variables of sample duration and parade size [file sj-docx-1-qjp-10.1177_17470218231155738.docx]

Supplementary Material for:

**Identifying unfamiliar voices: examining the system variables of sample duration and parade size**

Pautz Nikolas^1^

McDougall Kirsty^2^

Mueller-Johnson Katrin^2^

Nolan Francis^2^

Paver Alice^2^

Smith Harriet^1^

^1^Nottingham Trent University, UK

^2^University of Oxford, UK

***Corresponding Author:***

Pautz Nikolas

Nottingham Trent University - Psychology

Nottingham NG1 4FQ

UK

[nikolas.pautz@ntu.ac.uk](mailto:nikolas.pautz@ntu.ac.uk)

**Supplementary Material A**

**Multidimensional scaling analysis for speaker selection**

Three groups of 15 speakers were randomly selected from the *DyViS* database (*DyViS* groups1, 2 and 3), one group of 15 speakers was randomly selected from the *YorViS* database, and one group of 15 speakers from Bradford (*WYRED* 1) and another group of 15 speakers from Wakefield (*WYRED* 2) were randomly selected from the *WYRED* database. Except for *YorViS* where the database was too small to allow this, any particularly unusual-sounding speakers were omitted from the selection and further speakers selected randomly until each group contained 15 speakers. For each speaker, two short audio clips (labelled ‘utterance 1 (U1)’ and ‘utterance 2 (U2)’) of approximately three seconds were extracted from the mock interview studio-quality recordings. The subjects of all U1 and U2 samples were consistent across speakers (all U1 samples related to the speaker denying knowledge of a man named Robert Freeman; U2 speech involved the speaker denying having been at the Yewtree Reservoir on Wednesday evening). Within each 15-speaker group, each speaker was matched with all other speakers and with himself to form 120 pairings. Each pairing of speakers was represented by a U1 and a U2 (by random assignment). The order in which the two utterances were presented (U1, U2 or U2, U1) was determined by random assignment and allocated to one of two order blocks. Each listener participant had an equal chance of listening to either order block. The sequence list for each set was generated in random order on each occasion.

Participants were exposed to all possible voice utterances in random order during a ‘practice’ trial. An optional break was offered every 40-trials. The process was completed for the first *DyViS* group and the *YorViS* group in prior experiments in a lab environment (Nolan et al., 2013; McDougall (forthcoming)). The online platform “Gorilla” (Anwyl-Irvine et al., 2020) was used to present the stimuli to listeners for an additional two *DyViS* and two *WYRED* groups online. Online data collection was necessary due to the Covid-19 pandemic.

The online and lab-based procedures were largely the same, however, to ensure that participants who undertook the task online were using headphones and not loudspeakers, a headphone screening task (Woods et al., 2017) was implemented. In this task, participants were required to identify the softest tone correctly out of three options, at least four out of six times; it was not possible to identify the softest tone correctly using speakers. If a participant failed the headphone screener initially, they were allowed one additional attempt before being rejected from the experiment. Additionally, a progress bar was included in the online version of the task so that participants had an idea of the time left to complete the task.

The experiment was conducted one listener at a time in a quiet room for the lab-based data collection and the online participants were required to be in a quiet environment – the latter requirement was partially enforced via the headphone screener; the stimuli were played via headphones or earphones at a comfortable but clearly audible volume. Before beginning the online experiment, participants were asked to play a sample of static noise and calibrate their hardware volume so that it was clear, but comfortable.

Each listener was required to read a set of instructions explaining that they would be asked to compare a number of voices and assess their degree of similarity. Participants were required to listen to both voice pairs before making a similarity judgement. When the first voice utterance was played, a text appeared on the screening alerting the participant that this was ‘Voice 1’ and after a one-second gap, the second voice utterance played with a corresponding ‘Voice 2’ text message. After both utterances had been played, the next screen displayed the question ‘How similar are these voices?’ and, below, the numbers 1 (very similar) to 9 (very different). The listener was asked to mouse-click on a number to register their judgment and to move on to the next pair of voices. Before each pairing there was a silence of 1.5 seconds. A total of N=20 participants (age range: 17-42 years, approximately balanced for gender, speakers of English as a first language, based in Great Britain for most of their lives, no known speech or hearing difficulties) rated the utterances for each of the six sample groups.

After the similarity ratings had been collected, analyses using Multidimensional Scaling (MDS) were undertaken in SPSS to determine which ten out of each of the groups of 15 speakers were rated to be the most similar to each other (as per McDougall, 2013 and McDougall et al, 2015). For each group of 15 speakers, the speaker selected as the target was the one who yielded the shorter sum of Euclidean distances between himself and the nine speakers closest to him in the five-dimensional space generated by the MDS for that 15-speaker group. The five speakers farthest from the target were discarded. The fifth (middlemost) speaker out of the nine most similarly rated speakers was selected as the replacement foil. Thus, each original 15-speaker group gave a set of ten speakers for use in an experimental parade: the speaker with whom all other voices were compared was the target voice, and all other speakers were foils except for the middlemost speaker, who was used as the replacement foil used in target-absent parades (see Tables A2 and A3 for the complete speaker list).

Reference

Makowski, D., Ben-Shachar, M. S., Chen, S. H., & Lüdecke, D. (2019). Indices of effect existence and significance in the Bayesian framework. *Frontiers in Psychology, 10, Article* 2767. <https://doi.org/10.3389/fpsyg.2019.02767>

**Table A1**

*An overview of the sample and recording characteristics of the forensic databases.*

| Database | Age | Accent | Number of speakers | Tasks relevant to the present study | Quality and Recording Equipment |
| --- | --- | --- | --- | --- | --- |
| *DyViS* | 18-25 | Standard Southern British English (SSBE) | 100 | Included: 1) Simulated police interview, 2) telephone call with accomplice | Studio quality (.wav, 44.1kHz); sound-treated rooms; Marantz PMD670 portable; Sennheiser M464-K6, 20cm from participant’s mouth. |
| *YorViS* | 18-25 | York English | 21 | Included: 1) Simulated police interview, 2) telephone call with accomplice | Studio quality (.wav, 44.1kHz); sound-treated rooms; Recorded directly onto PC, via a TAC Scorpion 16-8-2 mixing desk, using an M-Audio 24/96 sound card; Neumann U87Ai condenser microphone (with pop shield), 25 cm from participant’s mouth. Sennheiser M464-K6, 20cm from participant’s mouth. |
| *WYRED* | 18-30 | West Yorkshire (Bradford, Kirklees, Wakefield) | 180 (60 for each area) | Included: 1) Simulated police interview, 2) telephone call with accomplice | Studio quality (.wav, 44.1kHz) sound-treated booths; PMD661 KMII Handheld Solid State Recorder; Sennheiser HSP 4, 2cm from mouth |

**Table A2**

*Speaker selection ordered from closest Euclidean distance to farthest Euclidean distance from the target speaker (Experiment 1, nine-voice parade).*

| Database target group | Database speaker number |
| --- | --- |
| *DyViS* 1 (SSBE) | **56**, 115, 112, 39, 25, 28, 60, 95, 65, 111 \| 53, 62, 88, 106, 118 |
| *DyViS* 2 (SSBE) | **23**, 87, 2, 50, 76, 4, 37, 31, 35, 32 \| 1, 11, 21, 47, 113 |
| *DyViS* 3 (SSBE) | **80**, 30, 46, 107, 99, 81, 6, 75, 96, 40 \| 19, 54, 58, 68, 69 |
| *WYRED* 1 (Bradford) | **185**, 187, 175, 170, 176, 132, 189, 157, 156, 135 \| 147, 167, 172, 174, 191 |
| *WYRED* 2 (Wakefield) | **166**, 152, 178, 127, 103, 138, 141, 158, 146, 145 \| 111, 112, 131, 143, 164 |
| *YorViS* (York) | **8**, 2, 10, 7, 1, 4, 18, 19, 16, 21 \| 11, 12, 17, 20, 22 |

*Note.* Target speakers are **bolded** and replacement speakers are underlined. Speakers listed after | represent discarded least-similar speakers.

**Table A3**

*Speaker selection ordered from closest Euclidean distance to farthest Euclidean distance from the target speaker (Experiment 2, six-voice parade).*

| Database target group | Database speaker number |
| --- | --- |
| *DyViS* 1 (SSBE) | **56**, 115, 112, 39, 25, 28, 60 \| 95, 65, 111, 53, 62, 88, 106, 118 |
| *DyViS* 2 (SSBE) | **23**, 87, 2, 50, 76, 4, 37 \| 31, 35, 32, 1, 11, 21, 47, 113 |
| *DyViS* 3 (SSBE) | **80**, 30, 46, 107, 99, 81, 6 \| 75, 96, 40, 19, 54, 58, 68, 69 |
| *WYRED* 1 (Bradford) | **185**, 187, 175, 170, 176, 132, 189 \| 157, 156, 135, 147, 167, 172, 174, 191 |
| *WYRED* 2 (Wakefield) | **166**, 152, 178, 127, 103, 138, 141 \| 158, 146, 145, 111, 112, 131, 143, 164 |
| *YorViS* (York) | **8**, 2, 10, 7, 1, 4, 18 \| 19, 16, 21, 11, 12, 17, 20, 22 |

*Note.* Target speakers are **bolded** and replacement speakers are underlined. Speakers listed after | represent discarded least-similar speakers.

**Supplementary Material B (Experiment 1, nine-voice parade)**

**Table B1**

*Model comparisons for accuracy data (Experiment 1, nine-voice parade).*

| Model | Δ$\hat{elpd}$ | ΔSE |
| --- | --- | --- |
| Main effect: target presence | 0.0 | 0.0 |
| Main effects: sample duration + target presence | -1.5 | 0.8 |
| Interaction: sample duration x target presence | -2.1 | 1.3 |
| Intercept Only | -8.9 | 4.1 |
| Main effect: sample duration | -10.4 | 4.1 |

*Note.* Models are ordered from the model with the highest predictive performance $\hat{elpd}$ (with standard error [SE]) in the top row. Δ$\hat{elpd}$shows the difference in the predictive performance (with standard error [ΔSE]) of the best fitting model with main effect of target presence compared to all remaining models. All models were run with 30,000 iterations and model convergence was confirmed by the Rubin-Gelman statistic ($\hat{R}$ = 1) (Gelman & Rubin, 1992).

**Table B2**

*Interaction model for accuracy data, 60s sample duration as reference (Experiment 1, nine-voice parade).*

| Parameter | MAP | 95% HDI | $\hat{R}$ | ESS | BF01 | BF10 | p (MAP) |
| --- | --- | --- | --- | --- | --- | --- | --- |
| 15s Sample Duration | -0.23 | [-1.08 – 0.66] | 1 | 53679 | 2.01 | 0.50 | 0.907 |
| 30s Sample Duration | -0.15 | [-1.01 – 0.73] | 1 | 55010 | 2.11 | 0.46 | 0.942 |
| Target-Present | 1.07 | [ 0.29 – 1.82] | 1 | 51850 | 0.06 | 14.4 | 0.027 |
| 15s duration × Target Presence | 0.61 | [-0.46 – 1.61] | 1 | 49946 | 1.05 | 0.95 | 0.556 |
| 30s duration × Target Presence | -0.02 | [-1.07 – 1.02] | 1 | 51341 | 1.9 | 0.53 | 0.999 |

*Note.* MAP = *maximum a posteriori* estimates (i.e., the mode of the posterior distribution); 95% HDI = 95% Highest Density Interval; $\hat{R}$ = Rubin-Gelman statistic, 1 = model convergence;
ESS = effective sample size; BF = Bayes Factor calculated using the Save-Dickey density ratio method (Wagenmakers et al., 2010); p (MAP) = Bayesian equivalent of the p-value, related to the odds that a parameter has against the null hypothesis (H_0_) (Makowski et al., 2019).

**Supplementary Material C (Experiment 2, six-voice parade)**

**Table C1**

*Model comparisons for accuracy data (Experiment 2, six-voice parade).*

| Model | Δ$\hat{elpd}$ | ΔSE |
| --- | --- | --- |
| Main effect: target presence | 0.0 | 0.0 |
| Main effects: sample duration + target presence | -1.4 | 1 |
| Interaction: sample duration x target presence | -2.4 | 1.1 |
| Intercept Only | -5.1 | 3.3 |
| Main effect: sample duration | -6.4 | 3.4 |

*Note.* Models are ordered from the model with the highest predictive performance (with standard error [SE]) in the top row. Δ$\hat{elpd}$ shows the difference in the predictive performance (with standard error [ΔSE]) of the best fitting model with main effect of target presence compared to all remaining models. All models were run with 30,000 iterations and model convergence was confirmed by the Rubin-Gelman statistic ( $\hat{R}$= 1) (Gelman & Rubin, 1992).

**Table C2**

*Interaction model for accuracy data, 60s sample duration as reference (Experiment 2, six-voice parade).*

| Parameter | MAP | 95% HDI | $\hat{R}$ | ESS | BF01 | BF10 | p (MAP) |
| --- | --- | --- | --- | --- | --- | --- | --- |
| 15s Sample Duration | 0.02 | [-0.79 – 0.83] | 1 | 56806 | 2.46 | 0.412 | 0.996 |
| 30s Sample Duration | -0.22 | [-1.08 – 0.59] | 1 | 57968 | 2.08 | 0.475 | 0.873 |
| Target-Present | 1.06 | [ 0.33 – 1.79] | 1 | 53689 | 0.04 | 23.90 | 0.015 |
| 15s duration × Target Presence | -0.27 | [-1.28 – 0.72] | 1 | 53765 | 1.68 | 0.589 | 0.844 |
| 30s duration × Target Presence | -0.20 | [-1.21 – 0.84] | 1 | 53757 | 1.8 | 0.563 | 0.938 |

*Note.* MAP = *maximum a posteriori* estimates (i.e., the mode of the posterior distribution); 95% HDI = 95% Highest Density Interval; $\hat{R}$ = Rubin-Gelman statistic, 1 = model convergence;
ESS = effective sample size; BF = Bayes Factor calculated using the Save-Dickey density ratio method (Wagenmakers et al., 2010); p (MAP) = Bayesian equivalent of the p-value, related to the odds that a parameter has against the null hypothesis (H_0_) (Makowski et al., 2019).

**Supplementary Material D – Pooled analysis**

Data were analysed using Bayesian mixed models (Gelman et al., 2014; McElreath, 2016) with accurate parade identifications scored as 1 and inaccurate identifications as 0 in a 3 (sample duration: 15s, 30s, 60s) x 2 (target presence: present or absent) factorial design. This analysis treated the 6 targets as a random factor. The 60s sample duration condition was treated as the reference category. Leave-one-out cross validation was used to evaluate model comparisons (Vehtari et al., 2017). The fitted models’ predictive performance was estimated as the sum of the expected log pointwise predictive density ($\hat{elpd}$) alongside its standard error (SE). A model with a difference in SE (ΔSE) equal to or greater than 5 is suggestive of better predictive performance (Vehtari et al., 2017). As the interaction model had a ΔSE of 0.7 (< 5) when compared to the target-presence only model (the model with the highest predictive capability), the interaction model was selected for inferential purposes.

**Table D1**

*Model comparisons for accuracy data (Combined data).*

| Model | Δ$\hat{elpd}$ | ΔSE |
| --- | --- | --- |
| Main effect: Target-Present | 0 | 0 |
| All Main Effects | -2 | 1.3 |
| 2-way Interactions | -5.5 | 1.6 |
| Null model | -14.7 | 5.3 |
| Main effect: Parade size | -15.5 | 5.4 |
| Main effect: Sample Duration | -15.8 | 5.4 |

*Note.* Models are ordered from the model with the highest predictive performance (with standard error [SE]) in the top row. Δ$\hat{elpd}$ shows the difference in the predictive performance (with standard error [ΔSE]) of the best fitting model with main effect of target presence compared to all remaining models. All models were run with 30,000 iterations and model convergence was confirmed by the Rubin-Gelman statistic ( $\hat{R}$= 1) (Gelman & Rubin, 1992).

**Table D2**

*Interaction model for accuracy data, 60s sample duration as reference (combined data from Experiments 1 and 2).*

| Parameter | MAP | 95% HD | $\hat{R}$ | ESS | BF_01_ | BF_10_ | p (MAP) |
| --- | --- | --- | --- | --- | --- | --- | --- |
| 15s Sample Duration | -0.07 | [-0.76 – 0.74] | 1 | 57454 | 2.61 | 0.383 | 0.998 |
| 30s Sample Duration | -0.15 | [-0.89 – 0.65] | 1 | 55098 | 2.49 | 0.402 | 0.965 |
| Target Present | 1.10 | [ 0.51 – 1.87] | 1 | 54877 | < .001 | > 100 | 0.003 |
| 6-voice Parade | 0.3 | [-0.41 – 1.00] | 1 | 55490 | 1.95 | 0.512 | 0.694 |
| 15s duration × Parade size | -0.16 | [-0.68 – 0.93] | 1 | 64470 | 2.21 | 0.453 | 0.913 |
| 30s duration × Parade size | -0.10 | [-1.00 – 0.67] | 1 | 67089 | 2.33 | 0.429 | 0.981 |
| Parade size × Target Presence | -0.17 | [-0.89 – 0.55] | 1 | 67399 | 2.46 | 0.407 | 0.900 |
| 15s duration × Target Presence | 0.10 | [-0.98 – 0.63] | 1 | 60888 | 2.34 | 0.428 | 0.954 |
| 30s duration × Target Presence | -0.16 | [-0.91 – 0.73] | 1 | 60786 | 2.18 | 0.459 | 0.908 |

*Note.* MAP = *maximum a posteriori* estimates (i.e., the mode of the posterior distribution); 95% HDI = 95% Highest Density Interval; $\hat{R}$ = Rubin-Gelman statistic, 1 = model convergence;
ESS = effective sample size; BF = Bayes Factor calculated using the Save-Dickey density ratio method (Wagenmakers et al., 2010); p (MAP) = Bayesian equivalent of the p-value, related to the odds that a parameter has against the null hypothesis (H_0_) (Makowski et al., 2019).

**Figure D1***. Cell means with 95% HDIs between speaker groups, combined data from Experiments 1 (nine voices) and 2 (six voices).*


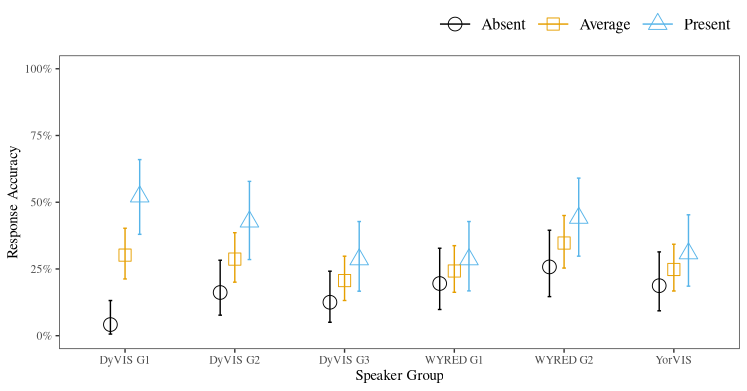


The cell means for by-speaker-group binary accuracy displayed in Figure D1 illustrates relatively stable accuracy when collapsing across target presence. Modeling binary accuracy by speaker-group with *DyViS* Group 1 as the reference category, we found negligible evidence to support the hypothesis that meaningful differences were present: *DyViS* 2: $\hat{\beta}$ = -0.03, HPDI: [-0.61 – 0.57], BF_10_ = 0.302; *DyViS 3*: $\hat{\beta}$ = -0.42, HPDI: [-1.07 – 0.17], BF_10_ = 0.837; *WYRED* *1*: $\hat{\beta}$ = -0.23, HPDI: [-0.86 – 0.35], BF_10_ = 0.425; *WYRED 2*: $\hat{\beta}$ = 0.23, HPDI: [-0.34 – 0.81], BF_10_ = 0.403; *YorViS*: $\hat{\beta}$ = -0.21, HPDI: [-0.82 – 0.38], BF_10_ = 0.397.

**Table D3**

*Model comparisons for accuracy data (Combined data, position effects).*

| Model | Δ$\hat{elpd}$ | ΔSE |
| --- | --- | --- |
| Main effect: Position | 0 | 0 |
| 2-way interaction | -1.3 | 0.9 |
| Null model | -11.9 | 4.7 |
| Main effect: Parade Size | -12.9 | 4.7 |

*Note.* Models are ordered from the model with the highest predictive performance (with standard error [SE]) in the top row. Δ$\hat{elpd}$ shows the difference in the predictive performance (with standard error [ΔSE]) of the best fitting model with main effect of target presence compared to all remaining models. All models were run with 30,000 iterations and model convergence was confirmed by the Rubin-Gelman statistic ( $\hat{R}$= 1) (Gelman & Rubin, 1992).
